# Combining In Vivo Two‐Photon and Laser Speckle Microscopy With the Ex Vivo Capillary‐Parenchymal Arteriole Preparation as a Novel Approach to Study Neurovascular Coupling

**DOI:** 10.1111/micc.70001

**Published:** 2025-01-07

**Authors:** Lowri E. Evans, Anna L. Gray, Katy R. Walsh, Thea G. E. Danby, Harry A. T. Pritchard, Stuart M. Allan, Alison M. Gurney, Adam S. Greenstein, Ingo Schiessl

**Affiliations:** ^1^ Geoffrey Jefferson Brain Research Centre, Manchester Academic Health Science Centre, Northern Care Alliance NHS Foundation Trust University of Manchester Manchester UK; ^2^ Division of Cardiovascular Sciences, School of Biological Sciences, Faculty of Biology, Medicine and Health University of Manchester Manchester UK; ^3^ Division of Infection, Immunity & Respiratory Medicine, Faculty of Biology, Medicine and Health University of Manchester Manchester UK; ^4^ Wellcome Centre for Cell‐Matrix Research, Lydia Becker Institute of Immunology and Inflammation, Faculty of Biology, Medicine and Health, Manchester Academic Health Science Centre University of Manchester Manchester UK; ^5^ Division of Neuroscience, School of Biological Sciences, Faculty of Biology, Medicine and Health University of Manchester Manchester UK; ^6^ Division of Pharmacy & Optometry, School of Health Sciences, Faculty of Biology, Medicine and Health University of Manchester Manchester UK

**Keywords:** cerebrovascular, in vivo imaging, myography, neurovascular

## Abstract

**Objective:**

Cerebral blood flow (CBF) decline is increasingly recognized as an area of importance for targeting neurodegenerative disorders, yet full understanding of the mechanisms that underlie CBF changes are lacking. Animal models are crucial for expanding our knowledge as methods for studying global CBF and neurovascular coupling in humans are limited and require expensive specialized scanners.

**Methods:**

Use of appropriate animal models can increase our understanding of cerebrovascular function, so we have combined chronic cranial windows with in vivo two‐photon and laser speckle microscopy and ex vivo capillary‐parenchymal arteriole (CaPA) preparations. Chronic cranial windows allow for longitudinal direct observation of the cerebral microvasculature and surrounding parenchyma while the CaPA preparation can assess capillary and arteriole function in isolation of the neuronal tissue.

**Results:**

Here, we found that extra‐dural cranial windows and related imaging protocols do not affect vascular function in the CaPA preparation. Cortical vessels from animals that have undergone imaging can therefore be taken to discover physiological alterations in the cerebral vasculature that contribute to any observed in vivo changes.

**Conclusion:**

This approach will enhance neurodegenerative research with the benefit of limiting animal usage.

## 1 Introduction

There is increasing awareness of the contribution of disrupted cerebral blood flow (CBF) in multiple neurodegenerative disorders, including Alzheimer's disease (AD) and Huntington's disease [1, 2, 3, 4]. Moreover, in AD and vascular dementia (VaD), there is evidence for dual deficits of reduced global CBF and impaired neurovascular function in both human and animal models [3, 5, 6, 7, 8, 9, 10, 11, 12]. Moreover, the magnitude of CBF reduction is associated with the degree of cognitive impairment [13]; therefore, understanding the mechanisms underlying CBF and its decline is key to preventing cognitive deterioration in dementia.

The precise mechanisms that control CBF in humans are difficult to study due to limited accessibility to the brain, the availability and resolution of imaging instruments, as well as ethical considerations. They are more tractable in animal models, although studies tend to be restricted to either in vivo or ex vivo analysis for studying CBF and neurovascular function. To address this issue, we sought to combine two cutting‐edge techniques used to investigate neurovascular function and its mechanisms: chronic cranial windows with two‐photon and laser speckle microscopy and the ex vivo capillary‐parenchymal arteriole (CaPA) preparation.

Cranial window implantation, where the murine skull is replaced with a glass coverslip, facilitates sufficient optical resolution of the cerebrovascular network to observe CBF down to individual capillaries. This allows the physiology of the full neurovascular unit to be examined in vivo, which can garner extensive understanding of dynamic cellular activities. Beyond this, chronic cranial windows enable long‐term cortical imaging [14], meaning that the effects of aging, disease development, and other altered states can be visualized to understand ramifications to the vasculature and surrounding neuronal tissue over time [15]. As the cranial window does not adversely affect the behavior of animals [16], in vivo imaging can also be used to determine mechanisms that correlate with behavioral changes.

Functional hyperemia in the brain is sustained by neurovascular coupling (NVC), whereby blood is diverted to active brain regions to ensure the metabolic demands of the neurons are met. Capillaries play a central role in detecting increases in neural activity, by sensing an increase of interstitial K^+^ ion concentration. This augments the activity of the inwardly rectifying K^+^ (K_IR_) channel, leading to capillary endothelial cell hyperpolarization [17]. The hyperpolarizing signal is rapidly propagated in a retrograde fashion to feeding arterioles, promoting dilation, and causing increased blood flow at the capillary bed that initially sensed the neuronal activity [17].

**FIGURE 1:**
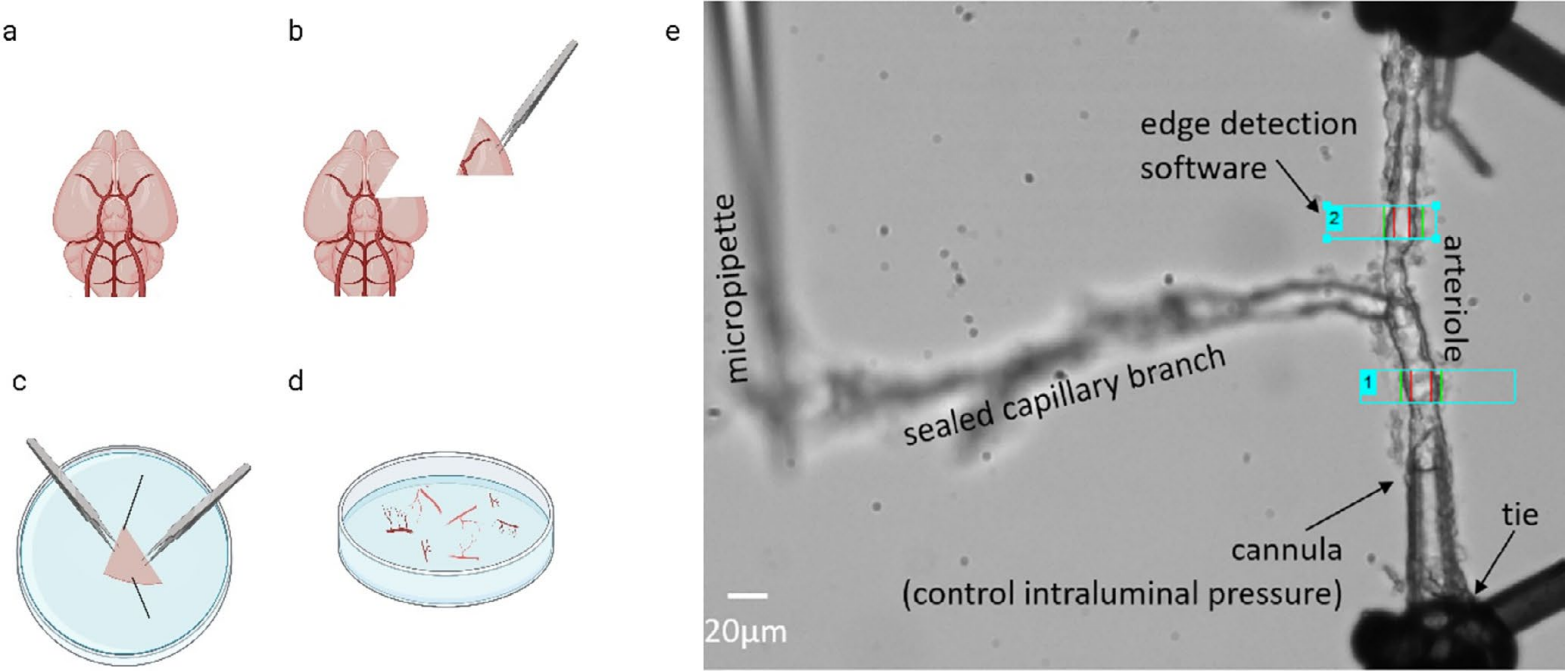
Capillary‐parenchymal arteriole preparation setup. Schematic of the capillary‐parenchymal arteriole preparation process. (a) Ventral view of isolated mouse brain, (b) brain ventral view with middle cerebral artery (MCA) section removed, (c) MCA section pinned in dissection dish ventral side down and tissue carefully prized apart using forceps to isolate the vasculature, (d) isolated parenchymal arterioles and attached capillaries, and (e) a parenchymal arteriole is cannulated at one end and tied off at the other, with the capillary branch sealed and held in place by a micropipette. The whole preparation is pressurized via the cannula and the internal lumen diameter recorded using edge‐detection software. The blue rectangles indicate windows in which the edges of the arteriole were detected and measured.

The ex vivo CaPA preparation dissects and cannulates penetrating parenchymal arteriole with a capillary branch still attached. The functional capillary is sealed off and stabilized to allow for a closed pressure loop (Figure 1). This living vascular bed is pressurized to physiological levels and allows experimentation on distinct vascular regions and mural cells without the disturbance of neuronal tissue [18, 19]. While the presence of multiple cell types can obscure the mechanistic properties of alterations in vivo, by removing the neuronal matter we can elucidate whether NVC changes have a vascular component.

In combining these two well‐established techniques, chronic cranial windows used for measurements of CBF and the ex vivo CaPA preparation, we can extend our ability to examine cerebral vascular function and dysfunction and add to our understanding of how vessels contribute to NVC regulation. If a change in vasoreactivity is observed in vivo, utilizing the CaPA preparation can probe the detail of these changes.

## 2 Methods

### 2.1 Mice

Adult male C57BL/6J obtained from Envigo were housed in pathogen‐free conditions on a 12‐h light/dark cycle, with ad libitum access to food and water. All experiments were approved by The University of Manchester Animal Welfare Ethical Review Board and conducted under project license authority from the UK Home Office, according to the Animals (Scientific Procedures) Act 1986. A group of nine mice were implanted with a cranial window for imaging and compared with a control group of eight mice that did not undergo any surgical procedure or imaging.

### 2.2 Cranial Window Implantation

**FIGURE 2:**
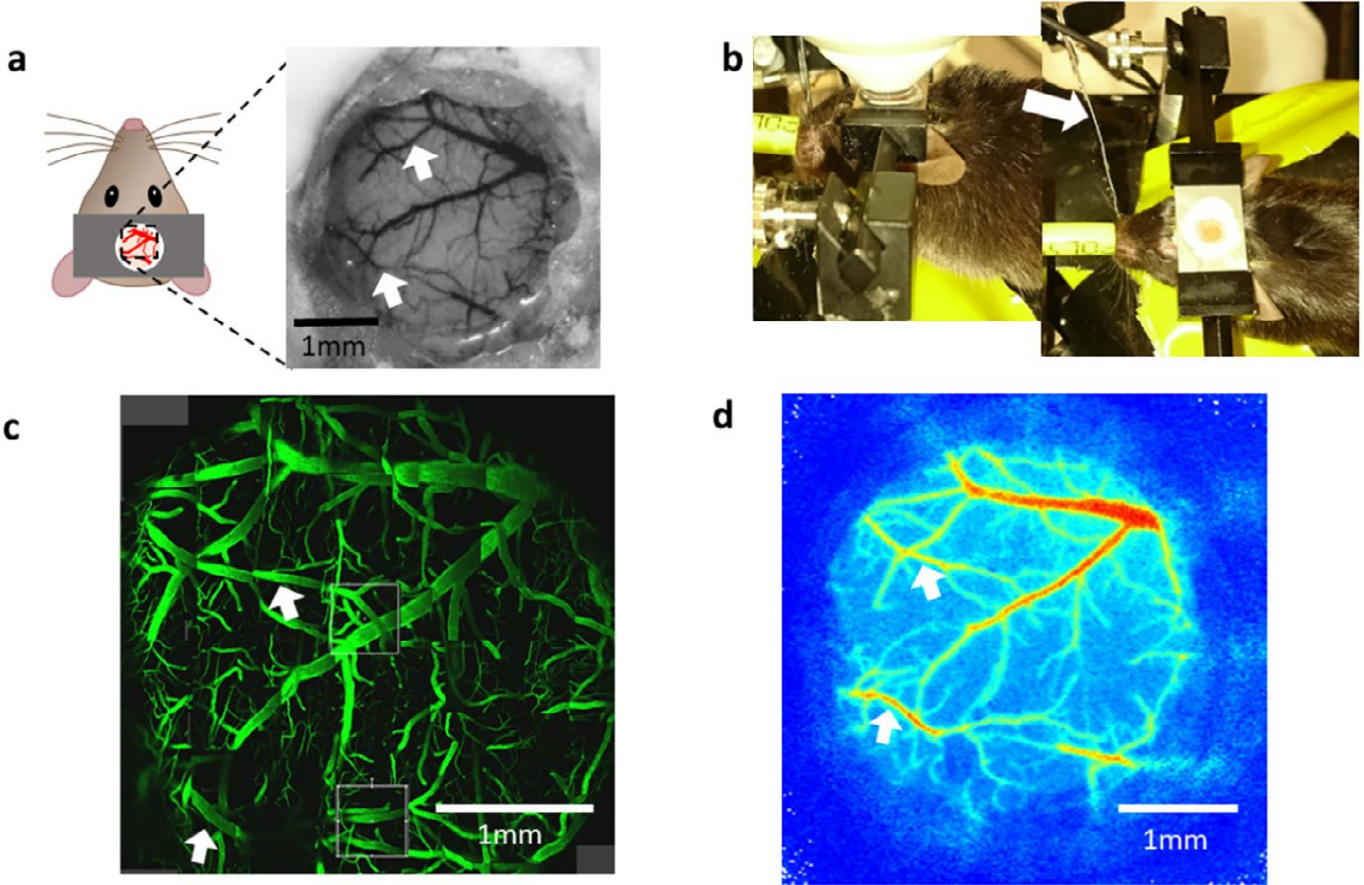
Experimental setup for imaging the vasculature under a cranial window with different imaging modalities in the same animal. (a) Cranial window implantation allows access to the pial vasculature in 3 mm diameter area. (b) Animal with cranial window in the two‐photon microscope with computer‐controlled whisker stimulation with objective (left) and with whisker stimulation highlighted with arrow (right). (c) The 3 mm diameter cranial window under the two‐photon microscope with FITC‐dextran injected into the tail vein. The visualization of this large area with sub‐micrometer resolution allows us to select several ROIs for 3D imaging, as indicated by gray boxes. (d) Vasculature under the cranial window in the laser speckle imaging system for the measurement of blood flow changes in response to somatosensory stimulation. White arrows show the location of the middle cerebral artery branches in each imaging modality.

Chronic cranial window implantation was completed as previously described [20, 21]. In short, 8–12‐week‐old mice weighing at least 24 g were anesthetized with 2.5% isoflurane in room air and positioned within a stereotactic frame with a heating blanket and temperature probe to ensure maintenance of body temperature at 37.5°C. The scalp was removed on top of both hemispheres to expose the cranium. The barrel cortex was marked on the bone using stereotaxic coordinates. A metal head plate (Narishige CP‐2, Japan) was mounted using dental cement (Sun Dental, Japan), for subsequent fixation within the two‐photon microscope setup. A circular piece of bone of 3 mm diameter over the barrel cortex was then carefully removed while leaving the dura intact. Lastly, a cranial window created from a 4 and 3 mm diameter circular coverslip (Warner Instruments, USA) glued together (thickness 0.2 mm) was secured with dental cement in place of the removed bone (Figure 2a). Animals were allowed to recover for a minimum of 1 week before the imaging sessions.

### 2.3 Two‐Photon Imaging

All animals in the imaging group were anesthetized throughout the experiment with 1.5% isoflurane in 100% oxygen. Animals were anesthetized for two‐photon microscopy for between 1.5 and 2.5 h. The two‐photon imaging session lasted for 1–1.5 h. The attached head plate was used to fix animals in place on the imaging platform which completely removes any head movement that could be introduced, for example by respiration. A temperature‐controlled heating blanket (Harvard Apparatus, UK) was used to maintain body temperature at 37.5°C. The two‐photon data sets were collected using a Leica SP8 multiphoton microscope in resonance mode with a Leica 25×/0.95 L HC Fluotar dipping objective at 800 nm excitation wavelength with a laser power of < 100 mW. To visualize the vasculature under the two‐photon microscope, 100 μL of 5 mg/mL 2 MDa fluorescein isothiocyanate–dextran (Sigma Aldrich, UK) was injected into the tail vein (Figure 2c). In order to record the changes in speed of blood flow, 1D line scans were placed in the vessel of interest and kymographs recorded with a speed of 16.000 lines per second over distances between 50 and 150 μm. These kymographs were then analyzed as previously described with the Matlab software provided by the authors (https://github.com/JiLabUCBerkeley/FACED2PFM‐vessel) [22, 23]. Under the described conditions, the analysis software was extremely robust and had no problems with extracting the flow speeds from the kymographs even in larger arterioles. Somatosensory stimulation was the same as for the laser speckle contrast imaging experiments.

### 2.4 Laser Speckle Contrast Imaging

Four of the mice that underwent two‐photon imaging were also imaged with the laser speckle contrast imager in a separate session (at least 3 days between the imaging sessions, Figure 2d). Mice were anesthetized with 2.5% isoflurane (Abbott, Berkshire, UK) and secured in a stereotaxic frame (World Precision Instruments, USA) positioned under a laser speckle contrast imager (Moor Instruments, UK). Throughout imaging, isoflurane anesthesia was maintained at 1.5% with body temperature monitored using a rectal thermometer and maintained at 37°C by an electric heating pad. Images were acquired at a frame rate of 1 Hz. To elicit a hemodynamic response, the whole whisker pad contralateral to the cranial window implantation was mechanically stimulated with a piezo‐ceramic actuator (Physik Instrumente Ltd., UK) (Figure 2b). The stimulation frequency was 10 Hz for a period of 6 s with an interstimulus interval of 14 s. The stimulation sequence was repeated 15 times. For the analysis, regions of interest were drawn over the visible surface vasculature and the average speed of flow extracted and then averaged over the 15 repetitions.

### 2.5 Capillary‐Parenchymal Arteriole Preparation

Cortical parenchymal arterioles were isolated as previously described [18, 19, 24]. In brief (at least 1 week after imaging for mice with cranial windows), following euthanasia by overdose of CO_2_ followed by exsanguination, the brain was removed and suspended in 3‐(N‐morpholino) propanesulfonic acid (MOPS)‐buffered saline (135 mM NaCl, 5 mM KCl, 1 mM KH_2_PO_4_, 1 mM MgSO_4_, 2.5 mM CaCl_2_, 5 mM glucose, 3 mM MOPS, 0.02 mM ethylenediaminetetraacetic acid (EDTA), 2 mM Na pyruvate, 10 mg/mL bovine serum albumin; pH 7.3 at 4°C). The middle cerebral arteries and surrounding tissue were dissected out and secured with pins in a Sylgard‐coated dish containing ice‐cold MOPS buffer (Figure 1). The tissue was stabilized in a central orientation and secured with dissecting pins. With forceps, the tissue was teased apart to locate parenchymal arterioles and attached capillaries, which were gently cut away from the tissue to ensure capillary attachment. Any neuronal tissue remaining was removed from the isolated vessels, which were kept on ice in the MOPS‐buffered solution. Arterioles were cannulated on borosilicate glass micropipettes in an organ chamber (University of Vermont Instrumentation and Model Facility, VT, USA). One end of the arteriole was occluded, and the end of the capillary branch was sealed by pressing it into the chamber coverslip with a glass micropipette (Figure 1). Vessels were pressurized to 40 mmHg using an arteriography system (Living Systems Instrumentation Inc., St. Albans, VT, USA). Lumen diameter was continuously monitored using a charge‐coupled device camera and edge‐detection software (IonOptix, Westwood, MA, USA). Vessels were continuously superfused at 4 mL/min (36.5°C) with gassed (5% CO_2_, 20% O_2_, and 75% N_2_) artificial cerebrospinal fluid (aCSF: 125 mM NaCl, 3 mM KCl, 26 mM NaHCO_3_, 1.25 mM NaH_2_PO_4_, 1 mM MgCl_2_, 4 mM glucose, 2 mM CaCl_2_; pH 7.3). Generation of at least 20% spontaneous myogenic tone of the arteriole when 40 mmHg was applied to the lumen (pressure‐induced constriction) [25] indicated preparation viability.

To investigate vessel function, reagents were applied locally by pressure ejection from a Picospritzer (Parker Hannifin, Ohio, USA) at 10 psi for 20s, through a filled glass micropipette placed either near the distal end of the capillary branch or at the arteriole. All experimental protocols began by stimulating the arteriole with 3 μM NS309 (Sigma‐Aldrich, MO, USA), an agonist at small (SK) and intermediate (IK) conductance Ca^2+^‐sensitive potassium channels. NS309 was then applied to the capillaries: as capillary endothelial cells do not express SK or IK channels, no arteriole dilation is expected. This protocol was used as a control to assess the spatial restriction of agents applied to capillaries by the Picospritzer. Preparations where no upstream arteriole dilation was observed in response to capillary NS309 stimulation were determined viable and the protocol continued. The next step was to apply aCSF containing 10 mM K^+^ via the Picospritzer at the capillaries and measure capillary‐to‐arteriole electrical signaling as an upstream arteriole dilation. 10 mM K^+^ was also applied to the arteriole to measure direct arteriole dilation. The maximum contractility of arterioles was assessed by exposing them to aCSF containing 60 mM K^+^. Passive diameter (PD) was measured in Ca^2+^‐free aCSF, prepared by omitting CaCl_2_ and adding 5 mM EGTA (See Supporting Information for calculations).

## 3 Results

### 3.1 In Vivo Imaging of the Hemodynamic Response Through the Cranial Window

**FIGURE 3:**
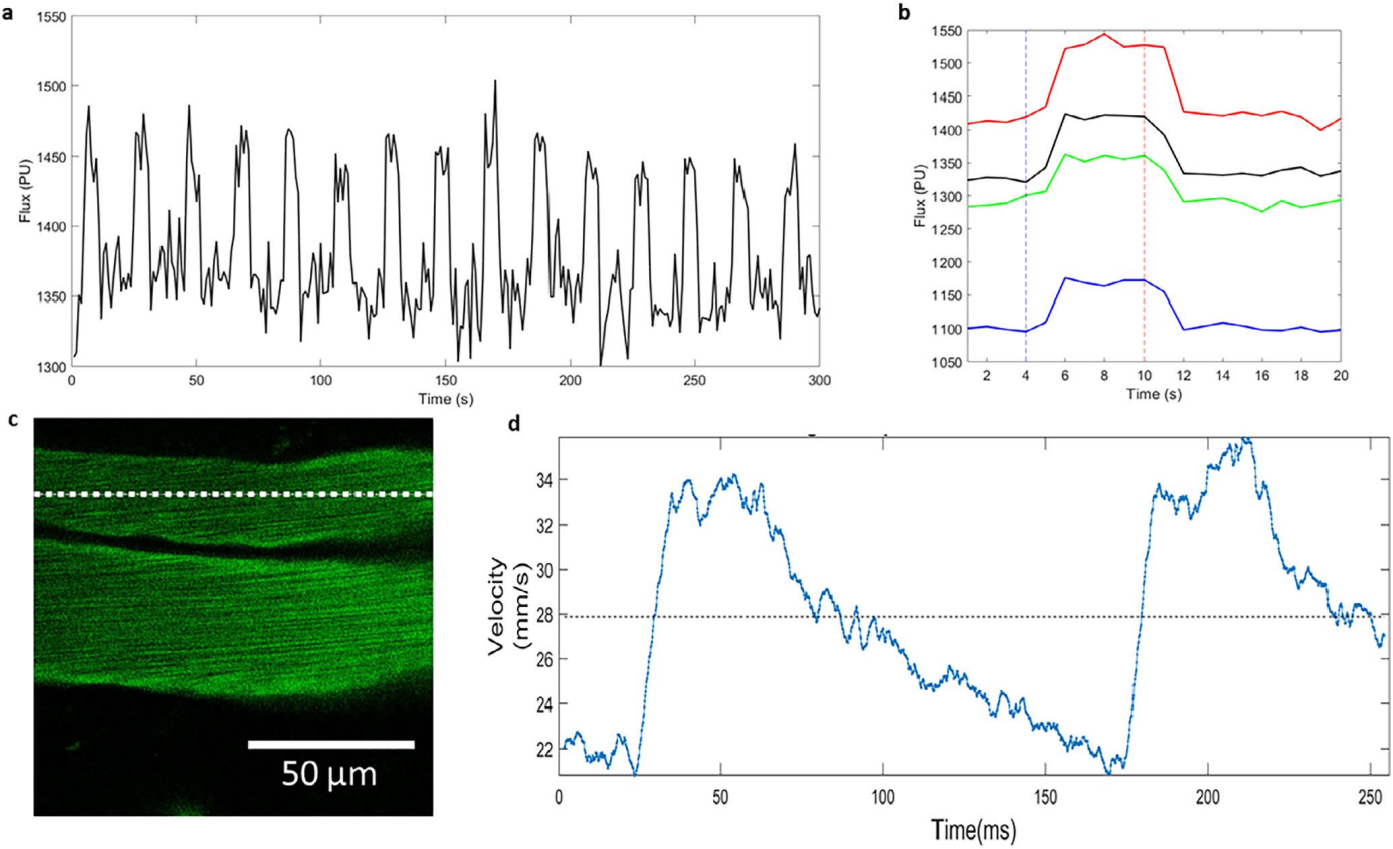
In vivo imaging of hemodynamic response through cranial window. (a) Changes in blood flow measured with the laser speckle system through the 3 mm diameter cranial window, with 15 increases of blood flow in response to mechanical whisker stimulation under isoflurane anesthesia clearly visible as increases in flux. (b) Average hemodynamic response for all four animals. The dotted blue vertical line at 4 s indicates the stimulus onset and the dotted red line at 10s indicated stimulus cessation. (c) High‐speed 1D line scan of a vessel during two‐photon imaging along the dotted line. Kymographs were recoded and analyzed to give local blood flow velocities as shown in (d).

In four of the animals that were later used for CaPA preparations, we imaged the hemodynamic response to mechanical whisker stimulation under isoflurane anesthesia through the cranial window with both the laser speckle imaging system and the two‐photon microscope on separate days. Imaging with the laser speckle system through the 3 mm diameter cranial window that was implanted over the barrel cortex gave reliable responses to the somatosensory stimulation in all animals (Figure 3a,b). While we observed a difference in the baseline flux value, the percentage change across all animals was 7.07 ± 1.54. The same animals were imaged a few days later with two‐photon imaging under the same levels of isoflurane anesthesia as used with laser speckle. For this protocol, we recorded the speed of blood flow via kymographs with the 1D line scans in vessels (Figure 3c) and were able to precisely visualize the changes to local blood flow following whisker stimulation. The short‐term changes shown in Figure 3d are mainly driven by heart beat and respiration and are often bigger than 50%. Because of this large variability, we were not able to detect the much smaller changes to the speed of blood flow triggered by the hemodynamic response in the current experiments.

### 3.2 Vascular Physiology Is Unaffected by Cranial Window Surgery and In Vivo Imaging

**FIGURE 4:**
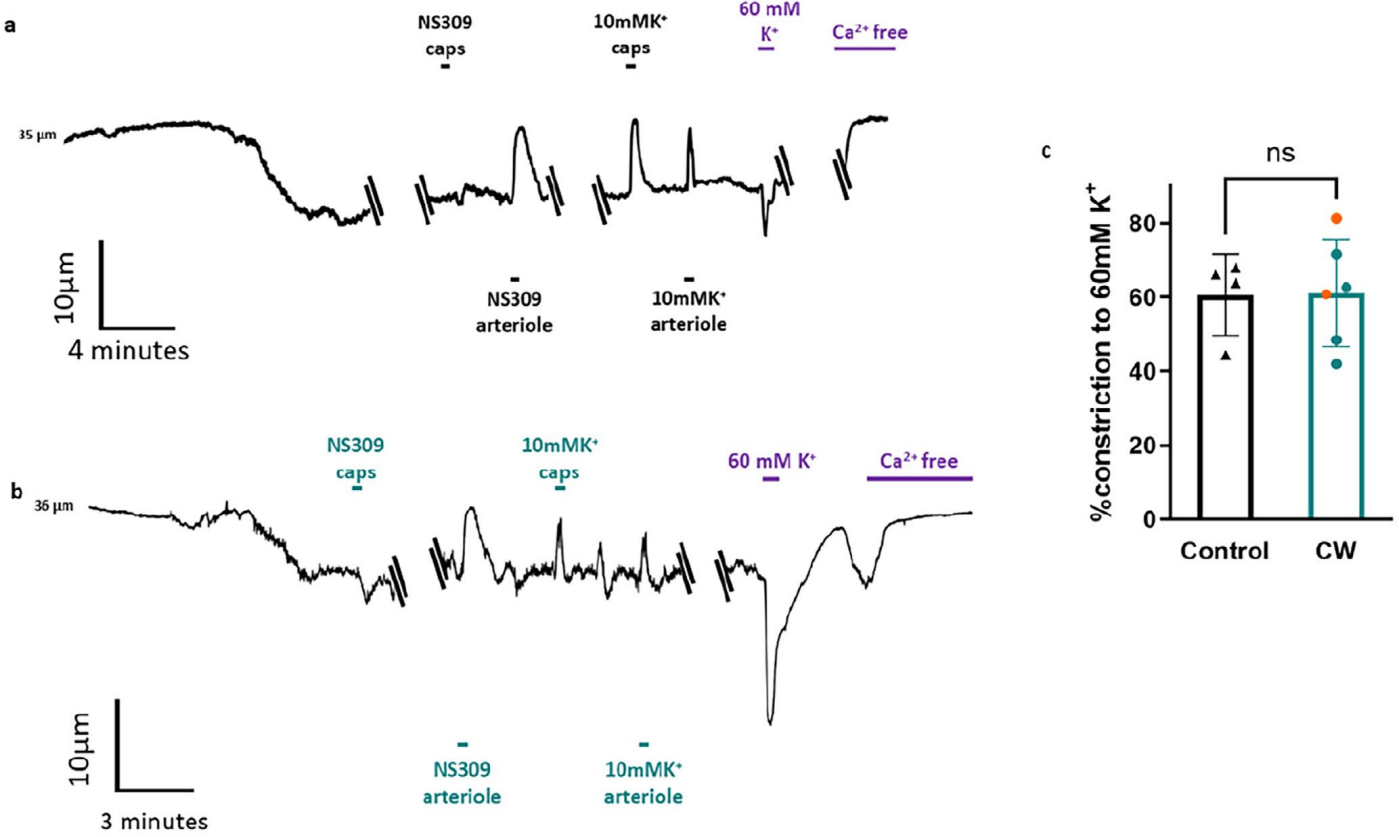
Capillary‐Parenchymal arteriole preparations retain contractility after in vivo imaging. Traces show continuous recordings of arteriole diameter from CaPA preparations isolated from control mice (a) and mice that were previously implanted with a cranial window and had undergone two‐photon and laser speckle imaging (b). Applications of 10 mM K^+^ and 3 μM NS309 to the capillaries (caps) are indicated above the traces and their direct application to the arteriole is indicated below the traces. Solution containing 60 mM K^+^ or Ca‐free solution was applied via bath perfusion as indicated in purple on both traces. (c) Average percent constriction developed in response to 60 mM K^+^ by control arterioles (*n* = 4) and imaged arterioles from mice with cranial windows (*n* = 6). Orange data points indicate imaged animals with both two‐photon and laser speckle. ns indicates no significant difference (*p* values > 0.05 determined by unpaired *t*‐test).

**FIGURE 5:**
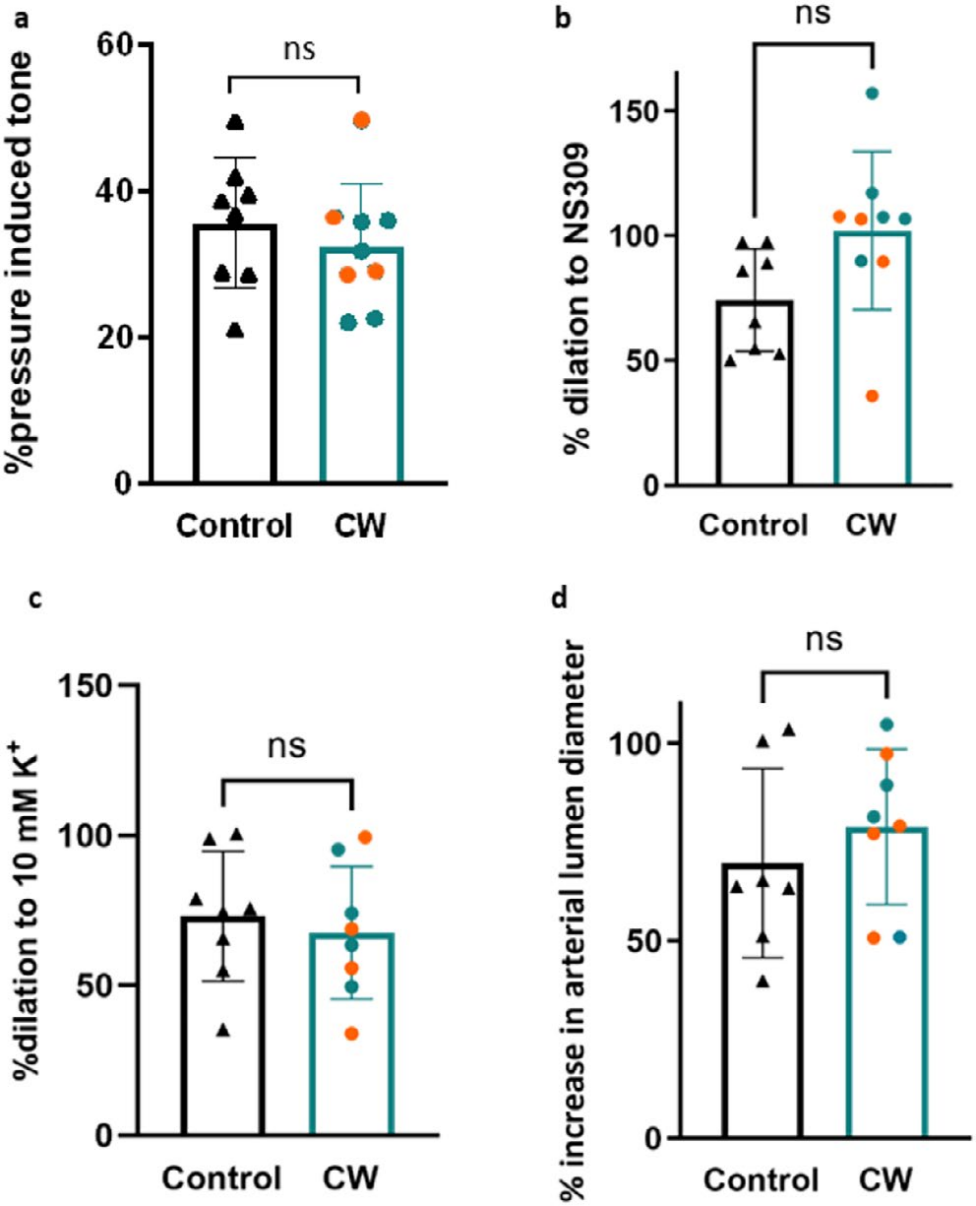
K channel‐dependent reactivity preserved in CaPA preparations after in vivo imaging. (a) Myogenic tone, plotted as percent pressure‐induced tone at 40 mmHg, is compared between control arterioles (*n* = 8) and imaged arterioles from mice with cranial windows (*n* = 8). Comparisons are also made between control and imaged arteriole diameter of vasodilation induced by 3 μM NS309 directly at the arteriole (b) or 10 mM K^+^ (c) applied directly to the arteriole (*n* = 8–9). (d) Arteriole dilation following the release of 10 mM K^+^ onto the attached capillaries in CaPA preparations from control (*n* = 7) and imaged (*n* = 8) arteries. Orange data points indicate imaged animals with both two‐photon and laser speckle. ns indicates no significant difference (*p* values > 0.05 determined by unpaired *t*‐test).

To determine whether vascular physiology is maintained following cranial window surgery and in vivo imaging, we measured the diameters of pressurized parenchymal arterioles. Recordings of vessel diameter (Figure 4a,b) show responses of arterioles from control and implanted and imaged mice to 3 μM NS309 or raised K^+^ concentrations, applied either directly to the arteriole or to the attached capillaries. Arterioles developed a similar maximum constriction (~60%) to 60 mM K^+^ whether they had been imaged through a cranial window or were isolated from control mice (Figure 4c). Contractile responses to physiological intraluminal pressure (40 mmHg), or myogenic tone, were also unaffected by cranial window surgery and imaging (Figure 5a). The functionality of key IK/SK ion channels was established by applying NS309. This had no effect on the arteriole when applied to capillaries as they do not express these channels (Figure 4a,b), but caused *a* > 50% increase in diameter when applied directly to the arteriole (Figure 5b). Importantly, there was no significant difference between the responses of control arterioles to NS309 and those that had been previously imaged through a cranial window. Finally, we studied the inwardly rectifying potassium channel K_IR_, which is expressed in both vascular smooth muscle cells and endothelial cells and mediates vasodilation in response to a small elevation in the extracellular K^+^ concentration [17]. This channel is key to regulating neurovascular coupling [17, 26, 27] and has been shown to be damaged in cerebral vessels in multiple disease states [9, 27, 28]. Cranial window implantation and imaging did not affect the ability of 10 mM K^+^ to dilate arterioles directly (Figure 5c) or via capillary‐to‐arteriole signaling (Figure 5d).

## 4 Discussion

We have shown that the combination of cranial window surgery with both two‐photon and laser speckle microscopy does not disrupt the integrity of arterioles and capillaries in the mouse cerebral cortex. Combining these in vivo imaging techniques with the ex vivo CaPA preparation is therefore a useful technical advancement for obtaining a greater understanding of the vascular physiological contributions to blood flow and NVC maintenance and regulation. Mice with the procedure and imaging had equivalent myogenic tone, comparable responses to the SK/IK channel agonist NS309, and capillary to arteriole signaling via the K_IR_ channel was not affected.

We were able to demonstrate that laser speckle contrast imaging under isoflurane anesthesia through the small 3 mm diameter cranial window delivered reliable responses to the mechanical whisker stimulation. Normally the ROI investigated with this imaging method is much larger as we have shown previously by imaging across both hemispheres of mice [29]. Therefore, it is important to know that pushing the laser speckle system to its limit with respect to zooming in on a small ROI does not prohibit a reliable measurement of the hemodynamic response. When imaging the same animals under the same conditions with the two‐photon system, we were able to record local blood flow changes with high temporal precision. As previously shown, these fluctuations in speed of flow regularly exceed 50% of the baseline flow speed in larger vessels [23]. Because of this large variability, the number of stimulus presentation in the current experiment was not big enough to detect the much smaller changes to the speed of flow in response to whisker stimulation after averaging the trials. This means that in order to detect these stimulus‐related changes, a much higher number of trials has to be recorded leading to recording times of over 30 min for a single location. While this might be feasible under anesthesia, it would be a big obstacle for awake behaving recording.

During two‐photon imaging, fluorescein isothiocyanate (FITC)‐dextran was used to image the vasculature. Large molecular weight dextran can be used as an indicator of blood‐brain barrier (BBB) permeability [30], which makes it an important tool in high‐resolution in vivo imaging after cranial window implantation. Moreover, previous work has shown that in vivo microscopy does not heighten inflammation [31]. While we did not take blood and measure inflammatory cytokines in this study, there were no signs of inflammation in any of the high‐resolution vascular images. However, in our previous studies, we have shown that our method of cranial window implantation, two‐photon and laser speckle imaging, did not increase inflammation or blood‐brain barrier permeability in the control groups that are equivalent to the imaged animals in this study [29, 31, 32].

Our measures of basic small arteriole and capillary physiology, through the CaPA preparation, further determine that cranial window implantations and subsequent imaging preserve the functionality of the vasculature. Our extra‐dural cranial window preparations also allow for longevity and clarity of windows, meaning multiple in vivo measurements can occur prior to termination and CaPA preparation.

These results also validate the use of isoflurane, which did not have a lasting impact on vascular physiology as measured by the CaPA preparation ex vivo. Throughout imaging sessions, a low dose (1.5%) of isoflurane was maintained. There is growing consideration of the use of isoflurane, as it has been reported to cause vasodilation [33, 34], yet remains widely used in in vivo blood flow and NVC investigations. As we observed no significant differences in the basic physiology of vessels, this suggests that any effects isoflurane may have had on vessels were short‐lived and we observed no long‐term physiological impact. While Isoflurane anesthesia reduces the size of the hemodynamic response to somatosensory stimulation, the response is still clearly present and measurable [35]. This reduction is happening in a dose‐dependent manner, and one of the advantages of the cranial window implantation at least 1 week ahead of the imaging sessions is that we can use low Isoflurane doses of about 1.5% as the animals experience no pain on that day. Mice also tolerate repeat Isoflurane anesthesia very well, and a recent study found no long‐term behavior and well‐being effects [36].

As the CaPA preparation requires the animal to be culled and the brain removed, the addition of in vivo imaging is useful for longitudinal or interventional studies. The CaPA preparation can examine whether any observed in vivo blood flow or neurovascular response changes correspond to alterations in vascular physiology. Longitudinal and interventional studies would further benefit from repeated vascular reactivity measures in vivo prior to myography, as drug effects may appear earlier than anticipated, or wane throughout treatment, which could only be observed using CaPA by greatly increasing the number of animals and culling these animals at multiple time points. This would increase time and cost, but importantly contradict the 3Rs principle aims to reduce, refine, and replace animal experiments [37]. Combining these two methodologies on the same animal not only allows for differences between animals to be explored, but it is hoped that many fewer animals will be required for experiments on neurovascular physiology.

### 4.1 Perspectives

There is a growing appreciation for multidisciplinary approaches to investigate CBF control and breakdown in disease states; however, complimentary methodologies are often thought not to be practical in the same animal. In principle, the combination of chronic cranial window implantation with in vivo imaging, plus the ex vivo CaPA preparation, can be utilized to better explain how the various vascular cell types contribute to CBF control in both health and disease states.

## Conflicts of Interest

The authors declare no conflicts of interest.

## Supporting information


Data S1.

## Data Availability

The data of this study are available from the corresponding author, Ingo Schiessl, upon reasonable request.
