# Supplementary material for: Combining In Vivo Two‐Photon and Laser Speckle Microscopy With the Ex Vivo Capillary‐Parenchymal Arteriole Preparation as a Novel Approach to Study Neurovascular Coupling
Source: Microcirculation. 2025 Jan 7;32(1):e70001. doi: 10.1111/micc.70001 (PMC11706670; doi:10.1111/micc.70001)
Supplement: Supplementary file 1 — Data S1. [file MICC-32-e70001-s001.docx]

Data was acquired with IonOptix software and then exported to Microsoft Excel. Myogenic tone percentage was calculated using the following equation:

$$Myogenic tone= \frac{Passive Diameter-Contracted Diameter}{Passive Diameter}x 100$$

Where ‘passive diameter’ is the luminal diameter following 0Ca2+ physiological saline solution.

Pharmacological agent effects on vasodilation/contraction were calculated using the following equations:

$$\% Dilation= \frac{Dilated Diameter-Baseline Diameter}{Passive Diameter-Baseline Diameter}x 100$$

$$\% Contraction= \frac{Baseline Diameter-Contracted Diamter}{Passive Diameter}x 100$$

Where ‘baseline diameter’ is the luminal diameter prior to drug addition and ‘dilated/contracted diameter’ is the luminal diameter following vessel response to the agent. Calculating the percent of contraction or dilation in regards to the passive diameter allows for normalisation for differences in vessel diameter.
